# Effects of Nutritional Interventions in the Control of Musculoskeletal Pain: An Integrative Review

**DOI:** 10.3390/nu12103075

**Published:** 2020-10-09

**Authors:** Carolina Rodrigues Mendonça, Matias Noll, Maria Clara Rezende Castro, Erika Aparecida Silveira

**Affiliations:** 1 Programa de Pós-Graduação em Ciências da Saúde, Universidade Federal de Goiás, Goiânia 74605-050, Goiás, Brazil; matias.noll@ifgoiano.edu.br (M.N.); mariaclara.recas17@gmail.com (M.C.R.C.); erikasil@terra.com.br (E.A.S.); 2 Instituto Federal Goiano, Campus Ceres, Ceres 76300-000, Goiás, Brazil

**Keywords:** diet therapy, fatty acids, obesity, inflammatory markers, fruit, muscle, skeletal

## Abstract

Food consumption has significant positive effects on an individual’s health status, including the reduction of symptoms associated with musculoskeletal pain. However, specific food groups indicated for the treatment of pain are not yet determined. Hence, this review aimed to analyze the effects of nutritional interventions with specific diets, oils and/or fatty acids, and foodstuffs in natura in the reduction of musculoskeletal pain. An integrative review was conducted in the following databases: Embase, PubMed, LILACS, and Google Scholar. Clinical trials written in English, Spanish, and Portuguese and published between 2000 and March 2020 were included in this review. Seventeen studies were included. Among these, a reduction of musculoskeletal pain with different types of nutritional interventions, such as vegan and Mediterranean diets and the consumption of blueberry, strawberry, passion fruit peel extract, argan oil, fish oil (omega-3), olive oil, and undenatured type II collagen and vitamin D gel capsules, was observed in 14 studies. Eight studies evaluated the profiles of several inflammatory markers, and of these, decreased interleukin (IL)-6, IL-1β, and tumor necrosis factor-α levels were observed in two studies. This review suggests that different nutritional interventions with specific diets, oils and/or fatty acids, and foodstuffs in natura reduce musculoskeletal pain, specifically in adults with osteoarthritis. Besides pain improvement, nutritional interventions, including the consumption of strawberry and vitamin D gel capsules, decrease the levels of several inflammatory markers.

## 1. Introduction

Musculoskeletal pain is a complex health problem that causes discomfort, results in poor quality of life, and affects millions of people worldwide [1,2,3]. This pain can arise in different musculoskeletal structures (e.g., muscles, bones, joints, ligaments and tendons, and periarticular tissues) [3,4,5]. Pharmacological and non-pharmacological interventions and surgical procedures are the mainstay treatments of musculoskeletal pain, and these treatments subsequently improve an individual’s physical function [6,7]. However, more recently, several noninvasive and non-pharmacological interventions used to reduce musculoskeletal pain have been investigated [3] such as nutritional interventions with specific diets or with the consumption of specific foods [8,9,10,11].

Some foodstuffs are potentially considered beneficial to reduce musculoskeletal pain including fruits, vegetables, and whole grains [12]. Several foods or substances with functional properties have been studied for their anti-inflammatory effects and/or their possible treatment of pain, such as omega-3 present in fish oil [13,14,15,16], olive oil [17,18], turmeric [19], and green tea [20]; resveratrol in grapes and wine [21]; capsaicin in pepper; and several flavonoids [22] in cabbage [23], cocoa [24], apple, and citrus fruits [25]. Furthermore, other studies have shown an association between the progression of osteoarthritis and vitamin D [26,27,28] and vitamin K [29] deficiencies and on the use of omega-3 and polyunsaturated fatty acids in the diet, positively affecting the biochemical composition of the cartilage of individuals with osteoarthritis [30]. Dietary habits are related to several chronic diseases and may be a major contributor to mortality and morbidity worldwide [31]. The balanced consumption of food has significant positive effects on an individual’s health status, body weight, and cardiovascular status [32,33,34]. Studies assessing the benefits of nutritional interventions indicate that some foods possibly have anti-inflammatory activities, neutralizing chronic inflammation and oxidative stress (an imbalance of free radicals and antioxidants in the body, which can lead to cell and tissue damage) [32], which are the main determining factors for chronic pain [12]. Additionally, some foods may regulate the immune system and pain perception, improving the functional loss associated with musculoskeletal conditions and providing a better quality of life [12,35,36,37]. However, each food has different properties and mechanisms that act to reduce pain and other musculoskeletal conditions, [11] and the mechanisms behind these interactions are still unclear and need to be further explored [11].

A systematic review on clinical practice guidelines for the evaluation and treatment of musculoskeletal pain described the main available recommendations to reduce musculoskeletal pain, including physical examination, monitoring of the patient’s progress, physical exercise, and manual therapy [38]. In this review article and in a broad literature review, consistent evidence regarding the nutritional treatment of chronic pain is not yet available. Thus, considering that (a) there is little knowledge about foods and diets in the treatment of musculoskeletal pain in humans, (b) there is a need to verify which foods are indicated for the treatment of pain [11], and (c) there are no dietary and nutritional recommendations in the national and international treatment protocols and consensus on the clinical treatment of musculoskeletal pain; this integrative review aimed to analyze the effects of nutritional interventions with specific diets, oils and/or fatty acids, and foodstuffs in natura in the reduction of musculoskeletal pain. In addition, our findings may stimulate further study in nutrition and pain management areas, as well as contribute to clinical practice, public health promotion, and existing gaps to be addressed in future studies.

## 2. Materials and Methods 

An integrative literature review was conducted based on the criteria of Whittemore and Knafl (2005) [39], focusing on the current scientific evidence in the area of nutrition and musculoskeletal pain. This review was conducted in the following six stages: formulation of the problem, establishment of the inclusion and exclusion criteria, definition of the information to be extracted from the articles identified and selected, analysis of information, interpretation of results, and presentation of the review [40]. To guide the search for scientific publications of intervention studies, the problem was formulated based on the PICO strategy [41], where P stands for people with musculoskeletal pain; I for nutritional interventions with specific diets, oils and/or fatty acids, and foodstuffs and other food supplements in natura in the treatment of pain; C for the control group with normal diet or the placebo group or other interventions for pain; and O for the expected outcome, which was the reduction of musculoskeletal pain.

The publications were collected by the principal investigator through an electronic search in the following databases: Embase, LILACS, PubMed, and Google Scholar. To perform the search, the Medical Subject Headings terms were used. The descriptors used for the searches were as follows: Musculoskeletal Pain, Pain, Osteoarthritis, Diet, Mediterranean, Diet Gluten-Free, Diet Fat-Restricted, Diet Vegetarian, Blueberry Plants, Plants Edible, Fruit, Vegetables, Fatty Acids, Fish Oils, and Olive Oil. The following filters were used for the PubMed search: Clinical Trial and Humans. The search strategy was combined and adapted for each of the databases using the Boolean operators (AND, OR, NOT or AND NOT).

The inclusion criteria were as follows: randomized clinical trials with nutritional interventions (interventions with specific diets, fruits and vegetables, or other foods in natura and interventions with the use of oils and/or fatty acids, vitamins, and other food supplements) in the treatment of musculoskeletal pain; studies that were written in English, Portuguese, or Spanish; and studies that were published between 2000 and March 2020. However, the following studies were excluded in this review: studies that combined diet and physical exercises or other types of therapies, considering that the combined use of two interventions can influence the results, subsequently preventing the identification of the intervention that resulted in the reduction of pain, studies assessing pain resulting from physical exercise and topical treatment, animal studies, theses, dissertations, case studies, review articles, editorials, letters to the editor, and duplicate studies.

Information on authors and year of publication, study population, place of study, follow-up period, interventions, outcomes assessed, and the main results of the clinical trials, including the reduction of pain and results of inflammatory markers when available, was collected. Due to the heterogeneity of the studies, the analysis was described according to the criteria of Whittemore and Knafl (2005) [39]. Thus, the studies were presented in subgroups based on the types of nutritional interventions. The Risk of Bias Assessment Tool from the Cochrane Collaboration will be used to assess the randomized clinical trial using RevMan Web [42]. This integrative literature review is based upon previously published studies. There are therefore no ethical concerns with regard to this study.

## 3. Results

**Figure 1:**
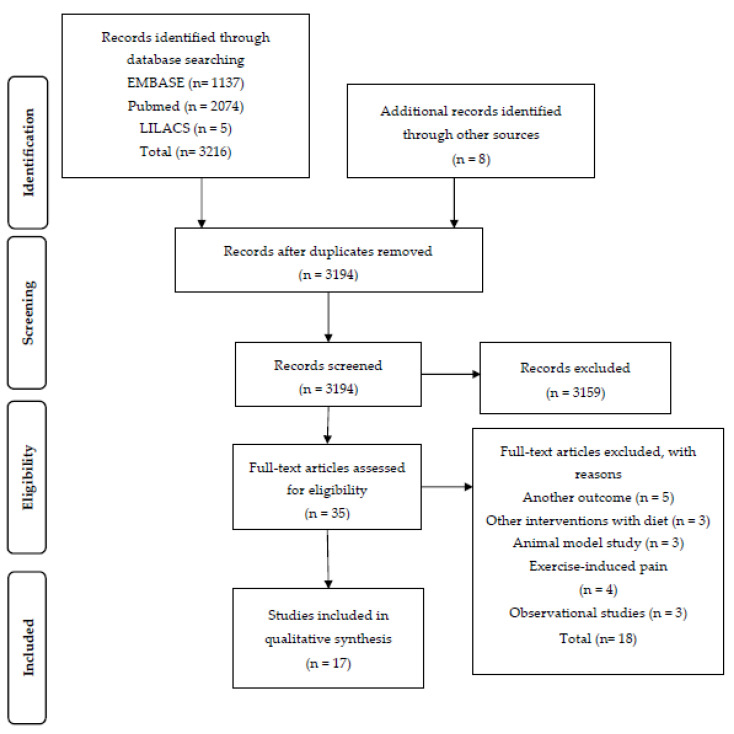
Study selection flowchart.

**Figure 2:**
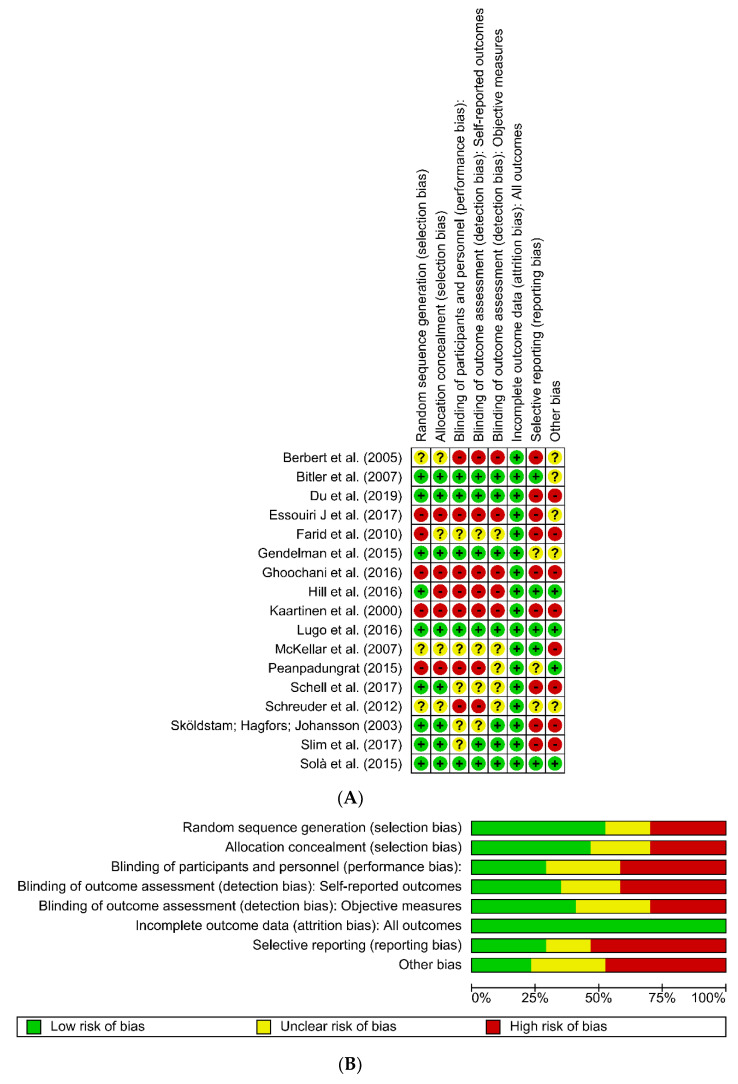
Assessment of risk of bias. (**A**) Summary of risk of bias for each trial (plus sign denotes low risk of bias; minus sign, high risk of bias; question mark, unclear risk of bias). (**B**) Risk of bias graph with each risk of bias item presented as percentages across all included studies.

A total of 3194 articles were identified, of which 35 were considered potentially relevant. In the final analysis, 17 studies were included (Figure 1). Of these, four studies assessed specific diets, four with nutritional interventions with fruits, five with the use of oils or fatty acids, and four with the use of other supplements. The studies were conducted in different countries, with the majority of the studies being conducted in Europe or North America. The full results of the included studies are presented in Table 1, Table 2, Table 3 and Table 4. Although the risk of general bias in the studies was low, some were identified as having a high risk of bias, as demonstrated in Figure 2, as they did not meet the following criteria: random sequence generation, allocation concealment, blinding of participants and personnel, blinding of outcome assessment, and selective reporting. 

### 3.1. Results of Nutritional Interventions

#### 3.1.1. Specific Diets

The effect of specific diets on musculoskeletal pain was demonstrated in four studies. There were a total of 284 participants, and two studies were randomized clinical trials [10,44]. Two studies assessed and compared the effects of a Mediterranean diet and a normal diet and demonstrated significant reductions in musculoskeletal pain with specific diets [43,44]. Another study demonstrated more significant improvements in pain with a vegan diet compared to an omnivorous control diet [45]. Pain reduction with the consumption of a gluten-free diet and a low-calorie diet was not observed [10]. Only one of these four studies investigated the levels of some inflammatory markers including C-reactive protein (CRP) and interleukin (IL)-6), and according to the result of this study, alterations in the levels of these markers were not observed [43] (Table 1). The follow-up period of these studies ranged from 6 to 24 weeks, and all studies were conducted in Europe.

#### 3.1.2. Nutritional Intervention with Fruits

Four studies evaluated the nutritional interventions with fruits, specifically the effects observed when blueberry [8], lyophilized strawberry powder [46], pomegranate juice [47], and passion fruit peel extract were consumed [48]. The studies had a total of 151 participants, and three were randomized and double-blind [8,46,48]. All studies compared the intervention group with the control or placebo group. Studies evaluating blueberry, strawberry, and passion fruit peel extract revealed that a significant reduction in musculoskeletal pain was observed after the consumption of blueberry, strawberry, and passion fruit peel extract [8,46,48].

Of the four studies, three evaluated the levels of some inflammatory markers [8,46,47], and according to the results of these studies, nutritional intervention with the consumption of strawberry demonstrated a significant reduction in the levels of IL-6 and IL-1B [46] (Table 2). The follow-up period in these studies ranged from 6 to 26 weeks. Two of the studies were conducted in the United States [8,46] and two in Iran [47,48].

#### 3.1.3. Nutritional Effects of the Use of Olive Oil, Omega-3, and Other Oils

Five studies [9,13,15,16,17] evaluated the nutritional effects of olive oil, omega-3, and other oils in the reduction of musculoskeletal pain, and all the nutritional interventions (argan oil in mL, fish oil [omega-3] in mL or g, or olive oil capsules) reported more significant reductions of pain in the treated group compared to the control group. A total of 510 participants were included in the randomized clinical trials. Only two studies evaluated the levels of some inflammatory markers, with one study evaluating the nutritional intervention with the consumption of olive oil capsules (assessing IL-B, IL-6, and IL-8 levels) [17] and another evaluating the nutritional intervention with the consumption of fish oil (assessing CRP level) [13]. According to the results of these two studies, significant alterations in the levels of some inflammatory markers evaluated were not observed. The follow-up period of the clinical trials ranged from 8 to 104 weeks (Table 3).

#### 3.1.4. Vitamin D and Other Food Supplements

Four studies evaluated the nutritional effects of vitamin D and other supplements based on the following interventions: consumption of undenatured type II collagen [49] and low-fat yoghurt supplemented with rooster comb extract (RCE) rich in hyaluronic acid [50] and rich in vitamin D in two studies [51,52]. There was a total of 428 participants in the three randomized double-blind trials [49,50,51]. The studies assessing the consumption of undenatured type II collagen and vitamin D demonstrated a reduction of musculoskeletal pain. Regarding the nutritional intervention involving the consumption of low-fat yoghurt supplemented with RCE, pain reduction was statistically insignificant between the placebo group and the intervention group [50]. Only two studies assessed the levels of some inflammatory markers [49,50,51], and according to the results of these studies, tumor necrosis factor (TNF)-α levels decreased in the group treated with vitamin D gel capsules [51]. The follow-up period of these studies ranged from 10 days to 25 weeks (Table 4).

#### 3.1.5. Pain Assessment Instruments

In the 17 articles analyzed, 5 different instruments were used for the assessment of musculoskeletal pain. Only one study [9] used two instruments, the WOMAC questionnaire and the Visual Analog Scale (VAS). The pain assessment instruments included the following: the multidimensional WOMAC questionnaire, used in seven studies [8,9,15,16,47,48,49]; Brief Pain Inventory-Short Form, used in one study [10]; Global Pain Scale, used in one study [43]; ICOAP Pain Questionnaire [46], used in one study; and VAS, the unidimensional scale, used in eight studies [9,13,17,44,45,50,51,52].

## 4. Discussion

This study aimed to analyze the effects of several nutritional interventions in reducing musculoskeletal pain. In this context, nutrition has scarcely been given attention, and its effects on pain have been underestimated but may become a key point in pain management [11]. According to the results of our study, different nutritional interventions have varying benefits in reducing musculoskeletal pain [8,9,13,15,16,17,44,45,46,48,49,51,52]. Out of the 17 studies assessing nutritional interventions, 14 demonstrated significant reductions in musculoskeletal pain and 2 reported a reduction in the levels of some inflammatory markers. Significant reductions in pain were not observed in only three studies [10,47,50].

### 4.1. Specific Diets

Two studies assessing a Mediterranean diet [43,44] and one study assessing a vegan diet [45] reported a reduction in musculoskeletal pain. This result is consistent with the result of a cohort comprising 4770 adults with osteoarthritis in which a Mediterranean diet was associated with a significant reduction of musculoskeletal pain [53]. Moreover, a recent meta-analysis investigated the effects of nutritional interventions on the severity and intensity of pain in individuals with chronic non-oncologic pain. Generally, it was observed that nutritional interventions (specific diets or consumption of one nutrient/food only) had significant effects on the reduction of pain [54].

Similar with this finding, Towery et al. (2018) [55] observed that the consumption of vegetable-based diets results in the reduction of musculoskeletal pain, while a balanced sugar diet (mono-, di-, and oligosaccharides) significantly reduces the symptoms of fibromyalgia [56]. These findings are possibly attributed to the higher concentrations of vitamins, minerals, and antioxidants provided by a balanced diet together with a lower intake of salt and animal fat, which acts positively on the outcome under analysis.

### 4.2. Nutritional Interventions with Fruits

Studies assessing nutritional interventions using different types of fruits such as blueberry [8], strawberry [46], and passion fruit peel extract [48] promoted a significant reduction in pain in individuals with osteoarthritis. This result is possibly attributed to the content of polyphenols present in blueberry and strawberries and flavonoids present in the passion fruit peel extract, which have anti-inflammatory effects, besides promoting anabolic effects in the cartilage, possibly explaining their activity against pain [57].

Consistent with these observations, a few studies have investigated the anti-inflammatory effects of polyphenols [58] on the cartilage [59] in individuals with osteoarthritis and observed that the daily intake of fruits, vegetables, carotenoids, and vitamin C [58] can improve pain, while vitamin D and K deficiencies are associated with loss of cartilage and injuries [59].

### 4.3. Effect of the Use of Olive Oil, Omega-3, and Other Oils

Nutritional interventions with oils or fatty acids, such as argan oil [9], olive oil capsules rich in polyphenols [17], and fish oil rich in omega-3, have been associated with a significant reduction in pain in individuals with knee osteoarthritis [13,15,16]. Studies on exercise-induced pain investigated the effects of supplementation with omega-3 derived from fish oil [60,61,62] and found a significant reduction of musculoskeletal pain after the consumption of fish oil. Two meta-analyses indicated that the consumption of omega-3 is possibly an adjuvant treatment in joint pain associated with rheumatoid arthritis, significantly reducing the administration of nonsteroidal anti-inflammatory drugs in patients with joint pain [63,64].

These results are possibly attributed to the beneficial effects of polyunsaturated fatty acids and omega-3 in osteoarthritis, considering that osteoarthritis is a metabolic condition in which lipids contribute to the pathophysiological degradation of the cartilage [30]. Moreover, omega-3 fatty acid supplementation increases the levels of E-series resolvins in synovial fluids, which are associated with pain reduction in patients with arthritis [65].

### 4.4. Vitamins and Other Food Supplements

Food supplements such as type II collagen [49] and vitamin D [51,52] showed significant reductions in musculoskeletal pain. Vitamin D consumption has been associated with cartilage regeneration in osteoarthritis, but the exact mechanism is not well defined [66]. Type II collagen consumption can reduce the inflammatory T-cell response and activate T-regulatory cells via its oral tolerance mechanism, which may reduce cartilage damage [67]. A recent systematic review evaluated the use of food supplements (unsaponifiable soy and avocado, capsaicin, turmeric, ginger, glucosamine, melatonin, polyunsaturated fatty acids, and vitamin D) in reducing musculoskeletal pain and noted that when food supplements are consumed as part of a balanced diet, they can aid in pain relief [68].

### 4.5. Strong Points

The results of this review are based on the data obtained from randomized clinical trials demonstrating that different nutritional interventions reduce pain and decrease the levels of some inflammatory markers. Thus, a balanced diet, rich in vitamins and minerals, may be a good option to reduce musculoskeletal pain considering that foods rich in vitamins and minerals contain antioxidants [48].

Considering the diverse set of studies, with different follow-up periods and pain assessment instruments used, assessing several nutritional interventions performed to reduce musculoskeletal pain, comprehensively demonstrating the effectiveness of nutritional interventions by performing a meta-analysis is impossible. However, this review has the following strength: this review comprises a large number of studies that assessed the effects of nutritional interventions on musculoskeletal pain in randomized clinical trials. 

### 4.6. Limitations

However, this study has the following limitations: we limited our search to studies carried out in the last 20 years, which may have limited the search; only few studies have investigated the levels of inflammatory markers; adverse effects were not assessed in this study; heterogeneity between the studies and types of nutritional interventions was observed; variation in the measures used to evaluate musculoskeletal pain was noted, and large variation in the follow-up period of clinical trials was evident in this study. We emphasize that it is necessary to assess the levels of several inflammatory markers in randomized clinical trials considering that these markers are possibly associated with the reduction in musculoskeletal pain. In addition, some studies had a small sample size and were not randomized or blinded.

### 4.7. Future Recommendations

Large-scale and robust randomized clinical trials, with longer follow-up periods, are required to confirm the effects of different nutritional interventions in reducing musculoskeletal pain. In addition, other additive or synergistic treatments as well as integrated approaches [69], such as physical exercises or physical therapy interventions and self-management and educational strategies, [69,70,71] could enhance the effects on pain reduction, and they should therefore be given attention [72]. Future studies should follow the CONSORT (Consolidated Standards Of Reporting Trials) [73] and ensure that appropriate methods are used for randomization, allocation, and assessment of results. Similarly, future studies should use standardized measures in pain evaluation, reporting the rates of adverse events, and should include the assessments of inflammatory markers. Additionally, further studies evaluating overweight, obesity, and other comorbidities that could affect the analysis of the pain reduction outcomes are required. High-quality studies assessing nutritional interventions performed in adults with musculoskeletal pain would be beneficial to researchers and clinicians in clinical practice and may improve the quality of life of people experiencing musculoskeletal pain. 

## 5. Conclusions

In summary, our findings indicate that food and nutritional interventions are considered beneficial in the treatment of pain and inflammatory conditions. For example, vegan and Mediterranean diets and the consumption of blueberry, strawberry, passion fruit peel extract, argan oil, fish oil (omega-3), olive oil, and undenatured type II collagen and vitamin D gel capsules reduce musculoskeletal pain, specifically in adults with osteoarthritis. Besides pain improvement, nutritional interventions, including the consumption of strawberry and vitamin D gel capsules, decrease the levels of several inflammatory markers including IL-6, IL-1β, and TNF-α.

## Figures and Tables

**Table 1:** Nutritional interventions with specific diets on musculoskeletal pain, other musculoskeletal manifestations, and inflammatory markers.

Authors/Year/ Country	Population	Type of Study	Follow-Up Period	Intervention	Outcomes Assessed	Pain Assessment Instrument Used	Main Results	Reduction of Pain
Slim et al. (2017) [10] Spain	*n* = 75 patients with fibromyalgia Age > 18 years	Randomized clinical trial	24 weeks	Group 1: Gluten-free diet (*n* = 35) Group 2: Low-calorie diet (*n* = 40)	Pain	Brief Pain Inventory-Short Form (BPI-SF)	There was a slight reduction in pain that did not differ significantly between the two study groups (*p* = 0.982)	No
McKellar et al. (2007) [43] United Kingdom	*n* = 130 women with rheumatoid arthritis Age: 30–70 years	Pilot study of dietary intervention	6 weeks	Group 1: Mediterranean diet (*n* = 75) Group 2: Healthy control diet (*n* = 55)	Pain Stiffness Inflammatory marker: CRP and IL-6	Global Pain Scale	Pain score was lower in group 1 (*p* = 0.011 and 0.049) than that in the control group, and morning stiffness (*p* = 0.041) was more predominantly observed in group 1 compared to the control group	Yes
Sköldstam; Hagfors; Johansson (2003) [44] Sweden	*n* = 51 Age: 33–75 years	Randomized, parallel study	13 weeks	Group1: Mediterranean diet (*n* = 26) Group 2: Control (regular diet) (*n* = 25)	Pain Swelling of the joints Stiffness	VAS	In group 1 there was an improvement in joint swelling (*p* = 0.001), and a reduction in pain (*p* = 0.006).	Yes
Kaartinen et al. (2000) [45] Finland	N = 28 with fibromyalgia Age: 34–62 years	Non-randomized clinical trial	13 weeks	Group 1: Vegan diet “living food”: uncooked foods, fruits, vegetables, mushrooms, nuts, seeds, legumes, and cereals (*n* = 18) Group 2: Control with omnivorous diet (*n* = 15)	Pain Stiffness	VAS	There was a reduction in pain observed on the visual analogue pain scale (*p* = 0.005) and joint stiffness (*p* = 0.001) in group 1.	Yes

IL-6, interleukin 6; CRP, C-reactive protein; VAS, Visual Analog Scale; a Daily intake of 1829 kcal, protein intake 71 g (16%-E), carbohydrate intake 276 g (53%-E), fat intake 63 g (31%-E), and no cholesterol.

**Table 2:** Nutritional interventions with fruits on the outcomes of musculoskeletal pain, other musculoskeletal manifestations, and inflammatory markers.

Authors/Year/ Country	Population	Type of Study	Follow-Up Period	Intervention	Outcomes Assessed	Pain Assessment Instrument Used	Main Results	Reduction of Pain
Du et al. (2019) [8] United States	*n* = 63 adults with self-reported symptomatic osteoarthritis Age: 45–79 years	Randomized, double-blind	17 weeks	Group 1: 40-g freeze-dried blueberry powder, in 20-g packs for daily consumption. Consumed twice a day (*n* = 27) Placebo group 2: Consumption of 40 g of “control” placebo powder daily, divided in 20-g packs, consumed twice a day (*n* = 22)	Pain Stiffness Inflammatory markers: Interleukin (IL)-1β, IL-6, IL-10, IL-13, TNF-α, MMP-3, MMP-13, and MCP-1	WOMAC questionnaire	There was a significant reduction of pain in the treatment group with blueberry (*p* < 0.05) There were no significant changes in the plasma concentrations of inflammatory markers in the treatment group (*p* > 0.05)	Yes
Schell et al. (2017) [46] United States	*n* = 17 adults diagnosed with osteoarthritis of the knee Average age: 57 ± 7 years	Randomized, double-blind	26 weeks	Group 1: 50 g of freeze-dried strawberry beverage consumed twice a day (*n* = a) Group 2: Placebo powder (*n* = a)	Pain Inflammatory markers: Interleukin (IL)-6, IL-1β, and MMP-3 and MMP-8	ICOAP Pain Questionnaire	Significant reduction of pain (all *p* < 0.05) in group 1 A significant reduction in the biomarkers interleukin (IL) -6, IL-1β after treatment with strawberry versus control (all *p* < 0.05).	Yes
Ghoochani et al. (2016) [47] Iran	*n* = 38 patients with osteoarthritis of the knee Age: 30–80 years	Randomized clinical trial	6 weeks	Group 1: pomegranate juice intervention (*n* = 19). Consumption of 200 mL without added sugar Group 2 (control): Usual lifestyle (*n* = 19)	Pain Stiffness Inflammatory markers: MMP-1, MMP-13	WOMAC questionnaire	Group 1 patients reported a significant reduction in stiffness (*p* = 0.00), but there was no reduction in pain scores in group 1 (*p* = 0.49) and group 2 (*p* = 0.13) There were significant differences between the two groups in relation to MMP-1 (*p* = 0.05) and MMP-13 (*p* = 0.02)	No
Farid et al. (2010) [48] Iran	N = 33 adults with osteoarthritis of the knee Age: 25–65 years	Randomized, double-blind, placebo-controlled study, with parallel group design	8 weeks	Group 1: Passion fruit peel extract (*n* = 17) Group 2 (placebo): pills with inactive ingredients without therapeutic activity and identical appearance (*n* = 16)	Pain	WOMAC questionnaire	There was a significant reduction in physical function after 30 days and pain after 60 days in group 1 (*p* < 0.001)	Yes

IL, interleukin; MCP-1, monocyte chemoattractant protein-1; MMP-3, matrix metalloproteinase-3; MMP-8, matrix metalloproteinase-8; MMP-13, matrix metalloproteinase-13; TNF, tumor necrosis factor; CRP, C-reactive protein; WOMAC, Western Ontario and McMaster Universities Arthritis Index; a, Not indicated; ICOAP, Intermittent and Constant Osteoarthritis Pain.

**Table 3:** Nutritional interventions with the use of olive oil, omega-3, and other oils on musculoskeletal pain, other musculoskeletal manifestations, and inflammatory markers.

Authors/Year/ Country	Population	Type of Study	Follow-up Period	Intervention	Outcomes Assessed	Pain Assessment Instrument	Main Results	Reduction of Pain
Essouiri J et al. (2017) [9] Morocco	*n* = 100 patients with osteoarthritis of the knee Average age: 58.24 ± 7.2 years	Randomized clinical trial	8 weeks	Group 1: argan oil to be consumed each morning (30 mL per day) (*n* = 51) Group 2: no treatment (*n* = 49)	Pain	VAS and WOMAC questionnaire	More significant reductions in pain were found by the VAS (*p* = 0.02) and WOMAC questionnaire (*p* < 0.0001) in group 1 compared to group 2	Yes
Hill et al. (2016) [15] Australia	*n* = 202 patients with osteoarthritis of the knee and pain Age: >40 years	Randomized clinical trial	104 weeks	Group 1. High dose of fish oil (omega-3 fatty acids 15 mL/day) (*n* = 101) Group 2. Low dose of fish oil (omega-3 fatty acids and canola oil 1: 9, 0.45 g) 15 mL/day. (*n* = 101)	Pain	WOMAC questionnaire	There was a greater reduction in pain scores at 2 years of follow-up in group 2 compared with group 1 There was no statistically significant difference between the two groups in a year of segment (*p* = 0.06)	Yes
Peanpadungrat (2015) [16] Thailand	*n* = 75 adults with osteoarthritis Age: 40–75 years	Randomized clinical trial	12 weeks	- Group 1. Without fish oil supplement (*n* = 25) - Group 2. Fish oil supplementation 1000 mg daily for 8 weeks (*n* = 25) - Group 3. Fish oil supplementation 2000 mg daily for 8 weeks (*n* = 25)	Pain Stiffness	WOMAC questionnaire	There was a significant reduction in pain and stiffness in groups 1 and 2 (*p* < 0.0001)	Yes
Bitler et al. (2007) [17] United States	*n* = 90 adults with osteoarthritis Age: 55–75 years	Double-blind, placebo-controlled randomized clinical trial Consumption of 2 capsules twice per day (100 mg)	8 weeks	Group 1: Olive oil capsules rich in polyphenols. (*n* = 43). Group 2: Placebo (*n* = 47).	Pain Inflammatory markers: IL-1β, IL-6, IL-8	VAS	There was a significant reduction of pain in the treatment group (*p* = 0.05)	Yes
Berbert et al. (2005) [13] Brazil	*n* = 43 patients Age: 20–73 years	Randomized clinical trial	12 and 24 weeks	Group 1. Placebo (soybean oil) (*n* = 13) Group 2. Omega-3 fish oil (3 g/d) (*n* = 13) Group 3. Fish oil - omega-3 fatty acids (3 g/d) and 9.6mL of olive oil (*n* = 17)	Pain Inflammatory marker: CRP	VAS	A more statistically significant reduction (*p* < 0.05) in the intensity of joint pain in groups 2 and 3 compared to group 1 was observed There was no statistically significant change in CRP	Yes

IL, interleukin; CRP, C-reactive protein; WOMAC, Western Ontario and McMaster Universities Arthritis Index; VAS, Visual Analog Scale.

**Table 4:** Nutritional interventions with other food supplements on musculoskeletal pain, other musculoskeletal manifestations, and inflammatory markers.

Authors/Year/ Country	Population	Type of Study	Follow-Up Period	Intervention	Outcomes Assessed	Pain Assessment Instrument	Main Results	Reduction of Pain
Lugo et al. (2016) [49] United States	*n* = 190 adults with osteoarthritis of the knee Age: 40–75 years	Randomized, double-blind, placebo-controlled clinical study	25 weeks	Group 1: undenatured type II collagen (40 mg) (*n* = 53) Group 2. placebo (*n* = 54)	Pain Stiffness Physical function Inflammatory markers: CRP, IL-6, MMP-3	WOMAC questionnaire	Significant reduction for all three WOMAC subscales in group 1: pain (*p* = 0.0003 vs. placebo), stiffness (*p* = 0.004 versus placebo), physical function (*p* = 0.007 vs. placebo)	Yes
Solà et al. (2015) [50] Spain	*n* = 80 adults with osteoarthritis of the knee Average age: 42.52 ± 13.16 years	Randomized, double-blind, placebo-controlled parallel study	12 weeks	Group 1. skimmed yogurt (125 mL d (1)) supplemented with 80 mg d (−1) of the crest of the rooster “roostercombextract (RCE) rich in hyaluronicacid” (*n* = 40) Group 2. Placebo-yogurt (*n* = 40)	Pain	VAS	There were no significant differences between the groups for the inflammatory markers	No
Gendelman et al. (2015) [51] Israel	*n* = 74 patients with musculoskeletal pain > 6 months Age: >18 years Follow-up period: 13 weeks	Randomized, double-blind and controlled study	13 weeks	Group 1. 4000 IU of vitamin D3 orally (4 gel capsules of 1000 units) (*n* = 36) Group 2. Placebo (*n* = 38) Both: regular pain reliever for 3 months	Pain Inflammatory markers: CRP, IL-6, TNF-α	VAS	There were no statistically significant differences between the intervention and control groups in relation to pain (p = not reported) TNFa levels dropped by 54.3% (after 6 weeks, *p* < 0.026) in the group treated with vitamin D	Yes
Schreuder et al. (2012) [52] Netherlands	*n* = 84 adults with musculoskeletal pain Age: 18–60 years	Randomized controlled study	6 weeks	Group 1. Vitamin D (150,000 IU of vitamin D (3) orally) (*n* = 44) Group 2. Placebo (*n* = 40)	Pain	VAS	Group 1 had significantly lower pain reduction than the placebo group (*p* < 0.001)	Yes

IL, interleukin; MMP-3, matrix metalloproteinase-3; TNF, tumor necrosis factor; CRP, C-reactive protein; WOMAC, Western Ontario and McMaster Universities Arthritis Index; VAS, Visual Analog Scale.

## References

[1] Clark S., Horton R. (2018). Low back pain: A major global challenge. Lancet.

[2] Hartvigsen J., Hancock M.J., Kongsted A., Louw Q., Ferreira M.L., Genevay S., Hoy D., Karppinen J., Pransky G., Sieper J. (2018). What low back pain is and why we need to pay attention. Lancet.

[3] Buchbinder R., Underwood M., Hartvigsen J., Maher C.G. (2020). The Lancet Series call to action to reduce low value care for low back pain: An update. Pain.

[4] Anandacoomarasamy A., Caterson I., Sambrook P., Fransen M., March L. (2008). The impact of obesity on the musculoskeletal system. Int. J. Obes..

[5] Arendt-Nielsen L., Fernández-de-Las-Peñas C., Graven-Nielsen T. (2011). Basic aspects of musculoskeletal pain: From acute to chronic pain. J. Man. Manip. Ther..

[6] Skelly A.C., Chou R., Dettori J.R., Turner J.A., Friedly J.L., Rundell S.D., Fu R., Brodt E.D., Wasson N., Winter C. (2018). AHRQ Comparative Effectiveness Reviews. Noninvasive Nonpharmacological Treatment for Chronic Pain: A Systematic Review.

[7] Foster N.E., Anema J.R., Cherkin D., Chou R., Cohen S.P., Gross D.P., Ferreira P.H., Fritz J.M., Koes B.W., Peul W. (2018). Prevention and treatment of low back pain: Evidence, challenges, and promising directions. Lancet.

[8] Du C., Smith A., Avalos M., South S., Crabtree K., Wang W., Kwon Y.H., Vijayagopal P., Juma S. (2019). Blueberries Improve Pain, Gait Performance, and Inflammation in Individuals with Symptomatic Knee Osteoarthritis. Nutrients.

[9] Essouiri J., Harzy T., Benaicha N., Errasfa M., Abourazzak F.E. (2017). Effectiveness of Argan Oil Consumption on Knee Osteoarthritis Symptoms: A Randomized Controlled Clinical Trial. Curr. Rheumatol. Rev..

[10] Slim M., Calandre E.P., Garcia-Leiva J.M., Rico-Villademoros F., Molina-Barea R., Rodriguez-Lopez C.M., Morillas-Arques P. (2017). The Effects of a Gluten-free Diet Versus a Hypocaloric Diet Among Patients With Fibromyalgia Experiencing Gluten Sensitivity-like Symptoms: A Pilot, Open-Label Randomized Clinical Trial. J. Clin. Gastroenterol..

[11] Elma Ö., Yilmaz S.T., Deliens T., Clarys P., Nijs J., Coppieters I., Polli A., Malfliet A. (2020). Chronic Musculoskeletal Pain and Nutrition: Where Are We and Where Are We Heading?. PM R.

[12] Rondanelli M., Faliva M.A., Miccono A., Naso M., Nichetti M., Riva A., Guerriero F., De Gregori M., Peroni G., Perna S. (2018). Food pyramid for subjects with chronic pain: Foods and dietary constituents as anti-inflammatory and antioxidant agents. Nutr. Res. Rev..

[13] Berbert A.A., Kondo C.R., Almendra C.L., Matsuo T., Dichi I. (2005). Supplementation of fish oil and olive oil in patients with rheumatoid arthritis. Nutrition.

[14] Hershman D.L., Unger J.M., Crew K.D., Awad D., Dakhil S.R., Gralow J., Greenlee H., Lew D.L., Minasian L.M., Till C. (2015). Randomized Multicenter Placebo-Controlled Trial of Omega-3 Fatty Acids for the Control of Aromatase Inhibitor-Induced Musculoskeletal Pain: SWOG S0927. J. Clin. Oncol..

[15] Hill C.L., March L.M., Aitken D., Lester S.E., Battersby R., Hynes K., Fedorova T., Proudman S.M., James M., Cleland L.G. (2016). Fish oil in knee osteoarthritis: A randomised clinical trial of low dose versus high dose. Ann. Rheum. Dis..

[16] Peanpadungrat P. (2015). Efficacy and Safety of Fish Oil in Treatment of Knee Osteoarthritis. J. Med. Assoc. Thai..

[17] Bitler C.M., Matt K., Irving M., Hook G., Yusen J., Eagar F., Kirschner K., Walker B., Crea R. (2007). Olive extract supplement decreases pain and improves daily activities in adults with osteoarthritis and decreases plasma homocysteine in those with rheumatoid arthritis. Nutr. Res..

[18] Rosillo M.A., Alcaraz M.J., Sanchez-Hidalgo M., Fernandez-Bolanos J.G., Alarcon-de-la-Lastra C., Ferrandiz M.L. (2014). Anti-inflammatory and joint protective effects of extra-virgin olive-oil polyphenol extract in experimental arthritis. J. Nutr. Biochem..

[19] Gaffey A., Slater H., Porritt K., Campbell J.M. (2017). The effects of curcuminoids on musculoskeletal pain: A systematic review. JBI Database Syst. Rev. Implement. Rep..

[20] Shen C.L., Cao J.J., Dagda R.Y., Chanjaplammootil S., Lu C., Chyu M.C., Gao W., Wang J.S., Yeh J.K. (2012). Green tea polyphenols benefits body composition and improves bone quality in long-term high-fat diet-induced obese rats. Nutr. Res..

[21] Shen C.L., Smith B.J., Lo D.F., Chyu M.C., Dunn D.M., Chen C.H., Kwun I.S. (2012). Dietary polyphenols and mechanisms of osteoarthritis. J. Nutr. Biochem..

[22] Staurengo-Ferrari L., Ruiz-Miyazawa K.W., Pinho-Ribeiro F.A., Fattori V., Zaninelli T.H., Badaro-Garcia S., Borghi S.M., Carvalho T.T., Alves-Filho J.C., Cunha T.M. (2018). Trans-Chalcone Attenuates Pain and Inflammation in Experimental Acute Gout Arthritis in Mice. Front. Pharmacol..

[23] Lauche R., Graf N., Cramer H., Al-Abtah J., Dobos G., Saha F.J. (2016). Efficacy of Cabbage Leaf Wraps in the Treatment of Symptomatic Osteoarthritis of the Knee: A Randomized Controlled Trial. Clin. J. Pain.

[24] Selmi C., Mao T.K., Keen C.L., Schmitz H.H., Eric Gershwin M. (2006). The anti-inflammatory properties of cocoa flavanols. J. Cardiovasc. Pharmacol..

[25] Shen W., Xu Y., Lu Y.H. (2012). Inhibitory effects of Citrus flavonoids on starch digestion and antihyperglycemic effects in HepG2 cells. J. Agric. Food Chem..

[26] Cao Y., Winzenberg T., Nguo K., Lin J., Jones G., Ding C. (2013). Association between serum levels of 25-hydroxyvitamin D and osteoarthritis: A systematic review. Rheumatology.

[27] Hirani V. (2012). Vitamin D status and pain: Analysis from the Health Survey for England among English adults aged 65 years and over. Br. J. Nutr..

[28] Zheng S., Tu L., Cicuttini F., Han W., Zhu Z., Antony B., Wluka A., Winzenberg T., Meng T., Aitken D. (2019). Effect of Vitamin D Supplementation on Depressive Symptoms in Patients With Knee Osteoarthritis. J. Am. Med. Dir. Assoc..

[29] Misra D., Booth S.L., Tolstykh I., Felson D.T., Nevitt M.C., Lewis C.E., Torner J., Neogi T. (2013). Vitamin K deficiency is associated with incident knee osteoarthritis. Am. J. Med..

[30] Masuko K., Murata M., Suematsu N., Okamoto K., Yudoh K., Nakamura H., Kato T. (2009). A metabolic aspect of osteoarthritis: Lipid as a possible contributor to the pathogenesis of cartilage degradation. Clin. Exp. Rheumatol..

[31] (2019). Health effects of dietary risks in 195 countries, 1990–2017: A systematic analysis for the Global Burden of Disease Study 2017. Lancet.

[32] Lobo V., Patil A., Phatak A., Chandra N. (2010). Free radicals, antioxidants and functional foods: Impact on human health. Pharmacogn. Rev..

[33] Marinho M.C.S., Hamann E.M., Lima A.C.d.C.F. (2007). Práticas e mudanças no comportamento alimentar na população de Brasília, Distrito Federal, Brasil. Rev. Bras. Saude Mater. Infant..

[34] Chin S.H., Huang W.L., Akter S., Binks M. (2020). Obesity and pain: A systematic review. Int. J. Obes..

[35] Dewell A., Weidner G., Sumner M.D., Chi C.S., Ornish D. (2008). A very-low-fat vegan diet increases intake of protective dietary factors and decreases intake of pathogenic dietary factors. J. Am. Diet. Assoc..

[36] Giugliano D., Ceriello A., Esposito K. (2006). The effects of diet on inflammation: Emphasis on the metabolic syndrome. JACC Cardiol. Oncol..

[37] Sabia M., Kalariya J. (2018). Nutrition and its effects on inflammation and chronic pain. J. Public Health Nutr..

[38] Lin I., Wiles L., Waller R., Goucke R., Nagree Y., Gibberd M., Straker L., Maher C.G., O’Sullivan P.P.B. (2020). What does best practice care for musculoskeletal pain look like? Eleven consistent recommendations from high-quality clinical practice guidelines: Systematic review. Br. J. Sports Med..

[39] Whittemore R., Knafl K. (2005). The integrative review: Updated methodology. J. Adv. Nurs..

[40] Soares C.B., Hoga L.A., Peduzzi M., Sangaleti C., Yonekura T., Silva D.R. (2017). Integrative review: Concepts and methods used in nursing. Rev. Esc. Enferm. USP.

[41] Milner K.A., Cosme S. (2017). The PICO Game: An Innovative Strategy for Teaching Step 1 in Evidence-Based Practice. Worldviews Evid. Based Nurs..

[42] Higgins J.P., Altman D.G., Gøtzsche P.C., Jüni P., Moher D., Oxman A.D., Savovic J., Schulz K.F., Weeks L., Sterne J.A. (2011). The Cochrane Collaboration’s tool for assessing risk of bias in randomised trials. BMJ.

[43] McKellar G., Morrison E., McEntegart A., Hampson R., Tierney A., Mackle G., Scoular J., Scott J.A., Capell H.A. (2007). A pilot study of a Mediterranean-type diet intervention in female patients with rheumatoid arthritis living in areas of social deprivation in Glasgow. Ann. Rheum. Dis..

[44] Skoldstam L., Hagfors L., Johansson G. (2003). An experimental study of a Mediterranean diet intervention for patients with rheumatoid arthritis. Ann. Rheum. Dis..

[45] Kaartinen K., Lammi K., Hypen M., Nenonen M., Hanninen O., Rauma A.L. (2000). Vegan diet alleviates fibromyalgia symptoms. Scand. J. Rheumatol..

[46] Schell J., Scofield R.H., Barrett J.R., Kurien B.T., Betts N., Lyons T.J., Zhao Y.D., Basu A. (2017). Strawberries Improve Pain and Inflammation in Obese Adults with Radiographic Evidence of Knee Osteoarthritis. Nutrients.

[47] Ghoochani N., Karandish M., Mowla K., Haghighizadeh M.H., Jalali M.T. (2016). The effect of pomegranate juice on clinical signs, matrix metalloproteinases and antioxidant status in patients with knee osteoarthritis. J. Sci. Food Agric..

[48] Farid R., Rezaieyazdi Z., Mirfeizi Z., Hatef M.R., Mirheidari M., Mansouri H., Esmaelli H., Bentley G., Lu Y., Foo Y. (2010). Oral intake of purple passion fruit peel extract reduces pain and stiffness and improves physical function in adult patients with knee osteoarthritis. Nutr. Res..

[49] Lugo J.P., Saiyed Z.M., Lane N.E. (2016). Efficacy and tolerability of an undenatured type II collagen supplement in modulating knee osteoarthritis symptoms: A multicenter randomized, double-blind, placebo-controlled study. Nutr. J..

[50] Sola R., Valls R.M., Martorell I., Giralt M., Pedret A., Taltavull N., Romeu M., Rodriguez A., Morina D., Lopez de Frutos V. (2015). A low-fat yoghurt supplemented with a rooster comb extract on muscle joint function in adults with mild knee pain: A randomized, double blind, parallel, placebo-controlled, clinical trial of efficacy. Food Funct..

[51] Gendelman O., Itzhaki D., Makarov S., Bennun M., Amital H. (2015). A randomized double-blind placebo-controlled study adding high dose vitamin D to analgesic regimens in patients with musculoskeletal pain. Lupus.

[52] Schreuder F., Bernsen R.M., van der Wouden J.C. (2012). Vitamin D supplementation for nonspecific musculoskeletal pain in non-Western immigrants: A randomized controlled trial. Ann. Fam. Med..

[53] Veronese N., Stubbs B., Noale M., Solmi M., Rizzoli R., Vaona A., Demurtas J., Crepaldi G., Maggi S. (2018). Adherence to a Mediterranean diet is associated with lower incidence of frailty: A longitudinal cohort study. Clin. Nutr..

[54] Brain K., Burrows T.L., Rollo M.E., Chai L.K., Clarke E.D., Hayes C., Hodson F.J., Collins C.E. (2019). A systematic review and meta-analysis of nutrition interventions for chronic noncancer pain. J. Hum. Nutr. Diet..

[55] Towery P., Guffey J.S., Doerflein C., Stroup K., Saucedo S., Taylor J. (2018). Chronic musculoskeletal pain and function improve with a plant-based diet. Complement. Ther. Med..

[56] Marum A.P., Moreira C., Tomas-Carus P., Saraiva F., Guerreiro C.S. (2017). A low fermentable oligo-di-mono-saccharides and polyols (FODMAP) diet is a balanced therapy for fibromyalgia with nutritional and symptomatic benefits. Nutr. Hosp..

[57] Kalt W., Cassidy A., Howard L.R., Krikorian R., Stull A.J., Tremblay F., Zamora-Ros R. (2019). Recent Research on the Health Benefits of Blueberries and Their Anthocyanins. Adv. Nutr..

[58] Han H., Chang C.B., Lee D.-C., Lee J.-Y. (2017). Relationship between total fruit and vegetable intake and self-reported knee pain in older adults. J. Nutr. Health Aging.

[59] Rayman M.P. (2015). Diet, nutrition and osteoarthritis. BMC Musculoskelet. Disord..

[60] Gray P., Chappell A., Jenkinson A.M., Thies F., Gray S.R. (2014). Fish oil supplementation reduces markers of oxidative stress but not muscle soreness after eccentric exercise. Int. J. Sport Nutr. Exerc. Metab..

[61] Tartibian B., Maleki B.H., Abbasi A. (2009). The effects of ingestion of omega-3 fatty acids on perceived pain and external symptoms of delayed onset muscle soreness in untrained men. Clin. J. Sport Med..

[62] Tsuchiya Y., Yanagimoto K., Nakazato K., Hayamizu K., Ochi E. (2016). Eicosapentaenoic and docosahexaenoic acids-rich fish oil supplementation attenuates strength loss and limited joint range of motion after eccentric contractions: A randomized, double-blind, placebo-controlled, parallel-group trial. Eur. J. Appl. Physiol..

[63] Goldberg R.J., Katz J. (2007). A meta-analysis of the analgesic effects of omega-3 polyunsaturated fatty acid supplementation for inflammatory joint pain. Pain.

[64] Lee Y.H., Bae S.C., Song G.G. (2012). Omega-3 polyunsaturated fatty acids and the treatment of rheumatoid arthritis: A meta-analysis. Arch. Med. Res..

[65] Barden A.E., Moghaddami M., Mas E., Phillips M., Cleland L.G., Mori T.A. (2016). Specialised pro-resolving mediators of inflammation in inflammatory arthritis. Prostaglandins Leukot. Essent. Fatty Acids.

[66] Garfinkel R.J., Dilisio M.F., Agrawal D.K. (2017). Vitamin D and Its Effects on Articular Cartilage and Osteoarthritis. Orthop. J. Sports Med..

[67] Gencoglu H., Orhan C., Sahin E., Sahin K. (2020). Undenatured Type II Collagen (UC-II) in Joint Health and Disease: A Review on the Current Knowledge of Companion Animals. Animals.

[68] Boyd C., Crawford C., Berry K., Deuster P. (2019). Conditional Recommendations for Specific Dietary Ingredients as an Approach to Chronic Musculoskeletal Pain: Evidence-Based Decision Aid for Health Care Providers, Participants, and Policy Makers. Pain Med..

[69] Kongsted A., Hartvigsen J., Boyle E., Ris I., Kjaer P., Thomassen L., Vach W. (2019). GLA:D^®^ Back: Group-based patient education integrated with exercises to support self-management of persistent back pain—Feasibility of implementing standardised care by a course for clinicians. Pilot Feasibility Stud..

[70] Palsson T.S., Boudreau S., Høgh M., Herrero P., Bellosta-Lopez P., Domenech-Garcia V., Langella F., Gagni N., Christensen S.W., Villumsen M. (2020). Education as a strategy for managing occupational-related musculoskeletal pain: A scoping review. BMJ Open.

[71] Kjaer P., Kongsted A., Ris I., Abbott A., Rasmussen C.D.N., Roos E.M., Skou S.T., Andersen T.E., Hartvigsen J. (2018). GLA:D^®^ Back group-based patient education integrated with exercises to support self-management of back pain-development, theories and scientific evidence. BMC Musculoskelet. Disord..

[72] Qaseem A., Wilt T.J., McLean R.M., Forciea M.A. (2017). Noninvasive Treatments for Acute, Subacute, and Chronic Low Back Pain: A Clinical Practice Guideline from the American College of Physicians. Ann. Intern. Med..

[73] Grant S., Mayo-Wilson E., Montgomery P., Macdonald G., Michie S., Hopewell S., Moher D. (2018). CONSORT-SPI 2018 Explanation and Elaboration: Guidance for reporting social and psychological intervention trials. Trials.

